# A new species of semiarboreal toad of the *Rhinella festae* group (Anura, Bufonidae) from the Cordillera Azul National Park, Peru

**DOI:** 10.3897/zookeys.673.13050

**Published:** 2017-05-12

**Authors:** Juan C. Cusi, Jiří Moravec, Edgar Lehr, Václav Gvoždík

**Affiliations:** 1 Departamento de Herpetología, Museo de Historia Natural, Universidad Nacional Mayor de San Marcos. Av. Arenales 1256, Jesus Maria, Lima 14, Peru; 2 Department of Zoology, National Museum, 19300 Praha 9, Czech Republic; 3 Department of Biology, Illinois Wesleyan University, Bloomington, IL 61701, USA

**Keywords:** Amphibia, phylogeny, *Rhinella acrolopha* group, *Rhinella lilyrodriguezae* new species

## Abstract

A new semiarboreal species of the *Rhinella festae* group is described from montane forests of the Cordillera Azul National Park between 1245 and 1280 m a.s.l. in the Cordillera Oriental, San Martín region, northern Peru. The new species is morphologically and genetically compared with members of the *Rhinella acrolopha* group (former genus *Rhamphophryne*) and members of the *R. festae* group. The new species is characterized by its large size (female SVL 47.1–58.3 mm, n = 4), eight presacral vertebrae, fusion of the sacrum and coccyx, long protuberant snout, snout directed slightly anteroventral in lateral view, cranial crests moderately developed, absence of occipital crest, presence of tympanic membrane, dorsolateral rows of small conical tubercles extending from parotoid gland to groin, hands and feet with long digits, fingers basally webbed and toes moderately webbed. Phylogenetically it is a member of the *R. festae* group which is most closely related to *R. chavin* and *R. yanachaga* from Peru. Morphologically the new species shares similarities with *R. tenrec* and *R. truebae*, members of the *R. acrolopha* group from Colombia.

## Introduction

The family Bufonidae Gray, 1825 comprises a clade of neobatrachian anurans commonly known as “true toads” of approximately 600 species and more than 50 genera (Frost 2016). *Rhinella* Fitzinger, 1826 (former part of the genus *Bufo*) is one of the most diverse bufonid genera currently composed of 91 species and broadly distributed throughout the Neotropical region (Pramuk 2006, Frost et al. 2006, Pereyra et al. 2015, Frost 2016). In Peru, the family Bufonidae has 54 species assigned to seven genera (AmphibiaWeb 2017): *Amazophrynella* (4), *Atelopus* (18), *Nannophryne* (2), *Rhaebo* (3), *Rhinella* (25), *Truebella* (2). Six similarity-defined groups within the genus *Rhinella* in South America are traditionally recognized based on anatomical and external characters, as follows: *Rhinella acrolopha, R. crucifer*, *R. granulosa*, *R. margaritifera*, *R. marina*, *R. spinulosa* and *R. veraguensis* groups (Martin 1972, Duellman and Schulte 1992, Grant and Bolívar-G. 2014)

The former genus *Rhamphophryne* Trueb, 1971, now a part of *Rhinella*, comprised several species of bufonid toads distributed in South American tropical forests. Most of the diversity of this genus occurs in montane forests, with nine species in northern Colombia and eastern Panama (*R. acrolopha* Trueb, 1971; *R. lindae* Rivero & Castaño, 1990; *R. macrorhina* Trueb, 1971; *R. nicefori* Cochran & Goin, 1970; *R. paraguas* Grant & Bolívar-G., 2014; *R. rostrata* Noble, 1920; *R. ruizi* Grant, 2000“1999”; *R. tenrec* Lynch & Renjifo, 1990; and *R. truebae* Lynch & Renjifo, 1990) and one species extending also to the upper Amazonian Basin of Ecuador (*R. festae* Peracca, 1904). From early on, the monophyly of the genus was not supported by Cannatella (1986) and Graybeal and Cannatella (1995). These authors noted that the only diagnostic feature of *Rhamphophryne* was the anteriorly ossified sphenethmoid forming a protuberant snout, which is variable in some species (*R. nicefori* and *R. rostrata*).

Recently, based on morphological and molecular data, the generic name *Rhamphophryne* was synonymized with *Rhinella* Fitzinger, 1826 by Chaparro et al. (2007) following results of Pauly et al. (2004), Frost et al. (2006) and Pramuk (2006). Grant and Bolívar-G. (2014) adopted the nomination *Rhinella acrolopha* group consisting of the species of the former genus *Rhamphophryne*, nevertheless, they did not formally propose delimitation of this species group. In a phylogenetic analysis based on the mitochondrial 16S rRNA gene of available species, Moravec et al. (2014) proposed a new species-group name, *Rhinella festae* group, for a clade of toads containing the following seven species: *R. chavin* (Lehr, Köhler, Aguilar & Ponce, 2001); *R. festae*; *R. macrorhina*; *R. manu* (Chaparro, Pramuk & Gluesenkamp, 2007); *R. nesiotes* (Duellman & Toft, 1979); *R. rostrata*; and *R. yanachaga* (Lehr, Pramuk, Hedges & Córdova, 2007). This monophylum is assumed to be integrated by certain species of the *R. acrolopha* group and some species of the *R. veraguensis* group (Pramuk 2006, Chaparro et al. 2007, Pramuk et al. 2008, Moravec et al. 2014, Grant and Bolívar-G. 2014). The *Rhinella veraguensis* group defined on morphological similarities by Duellman and Schulte (1992), modified by Padial et al. (2006) and Chaparro et al. (2007), is now recognized as a paraphyletic group. As a result, both *R. acrolopha* and *R. veraguensis* groups are artificial groupings lacking synapomorphies that define each species group. It is evident that comparison of molecular data of other *Rhinella* species is necessary to precise species composition of the individual species groups within the genus *Rhinella*. For example, a close relationship between [*R. festae* + [*R. macrorhina* + *R. rostrata*]] and [*R. chavin* + [*R. manu* + *R. nesiotes*]] was uncovered by Pyron and Wiens (2011). Grant and Bolívar-G. (2014) pointed out that sequences of *R. macrorhina* and *R. rostrata* were not included into the genetic analysis made by Moravec et al. (2014). However, we argue that the GenBank samples of “*R. macrorhina*” and “*R. rostrata*” used in studies of Van Bocxlaer et al. (2010) and Pyron and Wiens (2011) are probably both *R. macrorhina* and must be carefully evaluated due to lower quality of DNA sequences (see Taxon sampling for more details). Herein, we follow Moravec et al. (2014) and assign the below-described new species into the *Rhinella festae* group.

The Cordillera Azul National Park (herein CAZNP) located in the Cordillera Oriental of the Andes, Loreto, San Martín, Huánuco and Ucayali regions, is one of the most diverse natural protected areas in northern Peru. CAZNP has an extension of more than 13,000 km^2^ with an altitudinal range from 200 to 2400 m a.s.l. between the Huallaga and Ucayali rivers (INRENA 2006, Alverson et al. 2001). Rodríguez et al. (2001) conducted the first herpetological surveys of the CAZNP and recorded 58 species of amphibians and 24 species of reptiles from the basins of the rivers Pisqui and Pauya, Loreto. The recent discovery of new species of woodlizards (*Enyalioides azulae* Venegas, Torres-Carvajal, Durán & De Queiroz, 2013; and *E. binzayedi* Venegas, Torres-Carvajal, Durán & De Queiroz, 2013) and poison frogs (*Ranitomeya benedicta* Brown, Twomey, Pepper & Sanchez-Rodriguez, 2008; *R. summersi* Brown, Twomey, Pepper & Sanchez-Rodriguez, 2008; and *Ameerega yoshina* Brown and Twomey, 2009) within the CAZNP have increased the knowledge of the faunal diversity of this region. Most recently, Tasker and Twomey (2015) recorded 74 species of amphibians in the San Martín and Loreto regions of the CAZNP. As part of the aforementioned survey, Kaitlin Tasker and Juan C. Cusi conducted fieldworks in the surroundings of the Park Rangers Center N° 53 “Shapaja” at CAZNP, close to Tocache city in 2013, resulting in a discovery of a morphologically distinguishable *Rhinella* species, which was tentatively reported under the name Rhinella cf. festae by Tasker and Twomey (2015).

Here, a thorough comparison of this new bufonid from the Cordillera Azul National Park with other related *Rhinella* species is provided, its phylogenetic position based on DNA barcoding data elucidated, and the taxon formally described as a new species of the *Rhinella festae* group.

## Methods

**Figure 1.**
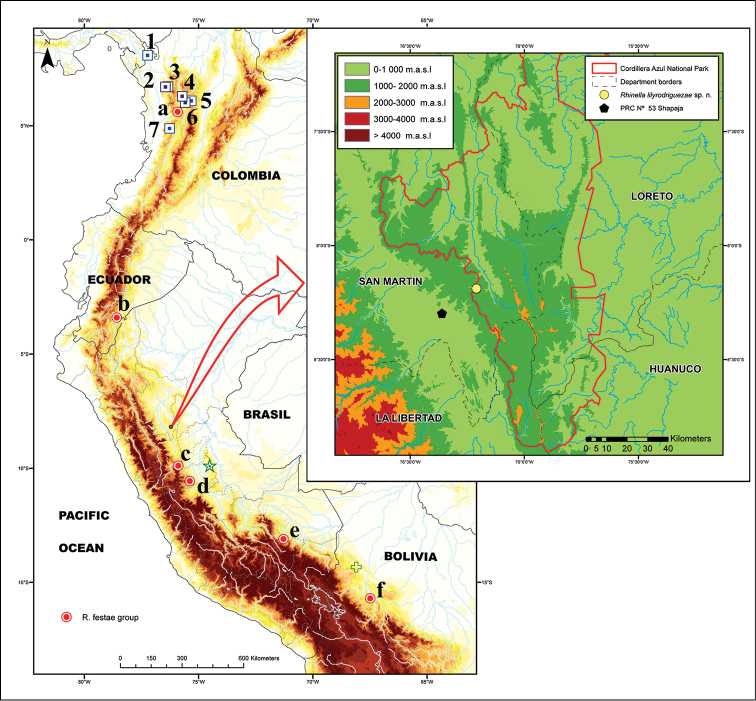
Map showing the type localities of *Rhinella lilyrodriguezae* sp. n. (○, yellow), selected species of the former genus *Rhamphophryne* (⊡, blue; with numbers **1** *R. acrolopha* **2** *R. tenrec* **3** *R. lindae* **4** *R. nicefori* **5** *R. rostrata* **6** *R. ruizi* **7** *R. paraguas*), species of the *Rhinella festae* group (⊙, red; with letters **a** *R. macrorhina* **b** *R. festae* **c** *R. chavin* **d** *R. yanachaga* **e** *R. manu*, f: Rhinella cf. nesiotes) and type localities of *Rhinella nesiotes* (★, green star) and *Rhinella tacana* (✙, cross, green). Abbreviation PRC: Park Rangers Center.

**Fieldwork and deposition of specimens.** Six specimens of the new species were collected inside the CAZNP (Fig. 1). All specimens were euthanized using an anesthetic 7.5% benzocaine gel on ventral surface of the individuals (McDiarmid 1994, Angulo et al. 2006). Tissue samples (liver and muscle pieces) were removed prior to preservation and stored in 96% ethanol, while specimens were fixed using 10% formalin and stored in 70% ethanol. All specimens were deposited at the herpetological collection of the Museo de Historia Natural, Universidad Nacional Mayor de San Marcos (**MUSM**), Lima, Peru.

The specimens were compared with species previously assigned either to the *Rhinella festae* group or the *R. acrolopha* group (see Appendix) and original species descriptions. The coordinates of the type and reference localities of species of the *R. acrolopha* group were obtained using Global Gazetteer version 2.3 (http://www.fallingrain.com/world/index.html). The geographic coordinates were based on the datum WGS84 and maps were designed using ArcGIS version 10.0.

**Morphological characters.** Morphometric measurements in millimeters (mm) were taken with a digital caliper Mitutoyo (nearest to 0.1 mm). Measurement abbreviations used throughout the text are:

**SVL** snout–vent length;

**HW** head width (at level of angle of jaw);

**HL** head length (from angle of jaw to tip of snout);

**ED** horizontal eye diameter;

**IOD** interorbital distance;

**EW** upper eyelid width;

**EL** eyelid length (upper eyelid length);

**IND** internarial distance;

**E–N** eye-nostril distance (straight line distance between anterior corner of orbit and posterior margin of external nares);

**NSD** nostril-snout distance;

**SL** snout length (between anterior corner of eye and tip of snout);

**FL** forearm length (between flexed elbow and proximal edge of palmar tubercle);

**HNDL** hand length (between proximal edge of palmar tubercle and tip of Finger III);

**FEML** femur length;

**TL** tibia length;

**FOOTL** foot length (distance from proximal margin of inner metatarsal tubercle to tip of Toe IV).

Fingers and toes are numbered preaxially to postaxially from I–IV and I–V, respectively. We determined comparative lengths of Toes III and V by adpressing both toes against Toe IV; lengths of fingers I and II were determined by adpressing the fingers against each other. Condition of the tympanum was assessed by visual examination under stereoscope. Specimens were sexed by examination of gonads and secondary sex characters. Ovarian eggs number and coloration were observed by dissection of a gravid female. The measurements of each egg were obtained of the maximum diameter, calculating the mean and standard deviation. Format of the description and diagnosis follows the standards of Trueb (1971), as modified by Grant (2000“1999”), and Grant and Bolívar-G. (2014).

Webbing formula follows Savage and Heyer (1967), Myers and Duellman (1982) and Savage and Heyer (1997). X-ray radiographs were obtained using a Carestream DirectView Vita CR computerized radiography system (http://www.carestream.es/computed-radiography/vita-cr-systems.html; 44 kV, 32 mAs) and were taken of the dorsal surface of each specimen collected. Images were edited with Adobe Photoshops CS6 for MacBookPro. Coloration in life of specimens was described based on digital photographs and field notes.

### Molecular phylogenetic analysis

**Taxon sampling.** Three specimens of the putative new species from the *Rhinella festae* group from the Cordillera Azul National Park were compared with the taxa from the dataset from Moravec et al. (2014), where the species group was established, supplemented by additional new material and sequences from GenBank. In particular, we included all taxa from the *R. festae* group available in GenBank for our genetic marker (16S rRNA), i.e. six taxa including one undescribed species “*Rhinella* sp. C” (Machado et al. 2016). We used only one sequence from two available of “*R. macrorhina*” and “*R. rostrata*” deposited in GenBank (Gluesenkamp *submitted 2001, unpublished*) due to their lower quality (based on comparison with other homologous sequences), and because they are most probably conspecific based on nucleotide sequences in overlapping DNA segments and information on origin of samples. Both samples were collected 0.5 km W of Medellin, Colombia by the same collector (P. Alberch), and are currently stored in the Museum of Vertebrate Zoology under the name “*Rhamphophryne macrorhina*” (voucher specimens/tissues: MVZ:Herp:231697/FC-13112; MVZ:Herp:150267/FC-13113). We therefore used only one sequence (AF375533) of “*R. rostrata*” and we name it *R. macrorhina* following David Cannatella’s identification (2008-12-11; see http://arctos.database.museum/guid/MVZ:Herp:231697). The same sample (GenBank sequences) was earlier used by Van Bocxlaer et al. (2010) and Pyron and Wiens (2011), and thus, their “*R. rostrata*” and “*R. macrorhina*” both represent *R. macrorhina* and the divergence is probably caused by errors in nucleotide sequences due to their lower qualities. Genetic information for *R. rostrata*, which might now be extinct (Stuart et al., 2008), is thus unavailable. The GenBank “*R. nesiotes*” based on the specimen UTA 53310 we name R. cf. nesiotes due to the very distant origin of this sample (Bolivia: La Paz, Caranavi, Serranía de Bella Vista; Pramuk and Lehr 2005) from the known range of the species (Peru: Huánuco: Reserva Comunal El Sira). In addition to the main focus on the *R. festae* group, we also investigated genetic identities of 14 samples from the *R. margaritifera*, *R. veraguensis*, and *R. marina* groups (serving also as outgroups), with a particular interest in the affinities of specimens morphologically resembling recently described *R. yunga* from a new locality, Rio Huatziroki (Pui Pui Protected Forest, Junín, Peru). More detailed information on the new material, and all analyzed samples from the *R. festae* group, is given in Table 1.

**Table 1.** Species, sample localities, museum numbers and GenBank accession numbers for DNA sequences used in the phylogenetic analysis of all samples of the *Rhinella festae* group, and new material of the *R. margaritifera*, *R. veraguensis* and *R. marina* groups. For the species group affiliation see Fig. 2.

Species	Locality	Museum No.	GenBank Accession No.	Reference
			16S Cytb	
*Rhinella lilyrodriguezae* sp. n.	Peru: San Martín, Bellavista, Alto Biavo, Cordillera Azul National Park	MUSM 32205	KY912598	This study
*Rhinella lilyrodriguezae* sp. n.	Peru: San Martín, Bellavista, Alto Biavo, Cordillera Azul National Park	MUSM 32206	KY912599	This study
*Rhinella lilyrodriguezae* sp. n.	Peru: San Martín, Bellavista, Alto Biavo, Cordillera Azul National Park	MUSM 32211	KY912600	This study
*Rhinella yanachaga*	Peru: Pasco, Oxapampa, Cordillera Yanachaga: Quebrada Yanachada, 2900 m	FMNH 282819	KF992148	Moravec et al. 2014
*Rhinella yanachaga*	Peru: Pasco, Oxapampa, Cordillera Yanachaga: Quebrada Yanachada, 2900 m	MUSM 31100	KF992149	Moravec et al. 2014
*Rhinella chavin*	Peru: Palma Pampa	MTD 43789	DQ158441	Pramuk 2006
*Rhinella* sp. C (= *Rhinella sp. acrolopha* group *sensu* Grant and Bolívar-G 2014)	--	--	KT221613	Machado et al. 2016
*Rhinella festae*	Ecuador: Pastaza, Petrolera Garza	KU 217501	DQ158423	Pramuk 2006
*Rhinella festae*	Ecuador: Napo, Estacion Biologica Jatun Sacha	QCAZ 18203	KR012624	dos Santos et al. 2015
*Rhinella macrorhina* (before “*R. rostrata*” in GenBank)	Colombia, Antioquia, 0.5 km W (by road) Medellin†	MVZ:Herp:231697 (FC-13112)	AF375533	Gluesenkamp, unpublished
*Rhinella macrorhina*‡	Colombia, Antioquia, 0.5 km W (by road) Medellin†	MVZ:Herp:150267 (FC-13113)	AF375532	Gluesenkamp, unpublished
Rhinella cf. nesiotes	Bolivia: La Paz, Caranavi, Serranía de Bella Vista	UTA 53310	DQ158478	Pramuk 2006
*Rhinella yunga*	Peru: Junín, area of Rio Huatziroki, buffer zone of the Pui Pui Protected Forest, 1950 m	MUSM 31950	KY912601	This study
*Rhinella yunga*	Peru: Junín, area of Rio Huatziroki, buffer zone of the Pui Pui Protected Forest, 2230 m	MUSM 31966	KY912602	This study
*Rhinella yunga*	Peru: Junín, area of Rio Huatziroki, buffer zone of the Pui Pui Protected Forest, 2075 m	NMP6V 75552	KY912603	This study
Rhinella cf. margaritifera	Peru: Junín, Ayte, buffer zone of the Pui Pui Protected Forest, 2007 m	MUSM 32713	KY912604	This study
Rhinella cf. margaritifera	Peru: Junín, Ayte, buffer zone of the Pui Pui Protected Forest, 2007 m	MUSM 32715	KY912605	This study
Rhinella cf. margaritifera	Peru: Junín, Ayte, buffer zone of the Pui Pui Protected Forest, 2007 m	IWU 334	KY912606	This study
Rhinella cf. margaritifera	Peru: San Martín, Rioja, Pardo Miguel, Alto Mayo Protected Forest	MUSM 34237	KY912607	This study
Rhinella cf. margaritifera	Peru: San Martín, Rioja, Pardo Miguel, Alto Mayo Protected Forest	MUSM 34238	KY912608	This study
Rhinella cf. margaritifera	Bolivia: Polpebra	CBF 5800	KY912609	This study
Rhinella cf. leptoscelis	Peru: Junín, area of Rio Bravo, buffer zone of the Pui Pui Protected Forest, 1721 m	MUSM 32726	KY912610	This study
Rhinella cf. leptoscelis	Peru: Junín, area of Rio Bravo, buffer zone of the Pui Pui Protected Forest, 1721 m	PE 008A	KY912611	This study
Rhinella cf. leptoscelis	Peru: Junín, area of Rio Bravo, buffer zone of the Pui Pui Protected Forest, 1721 m	PE 008B	KY912612	This study
Rhinella cf. leptoscelis	Peru: Junín, area of Rio Bravo, buffer zone of the Pui Pui Protected Forest, 1721 m	PE 008C	KY912613	This study
*Rhinella poeppigii*	Perú: Junin, La Merced, Pampa del Carmen, old swimming pool	MUSM 32746	KY912614	This study

† Database: http://arctos.database.museum ‡ This sequence was not included into our analysis. See Taxon sampling.

**Laboratory protocol and bioinformatics.** A fragment of the mitochondrial 16S rRNA gene (~ 550 bp) was targeted. DNA extraction, PCR amplification and sequencing followed the methods described in Moravec et al. (2009, 2014). The computational analysis followed the procedure and methodological approach, including used software, of Moravec et al. (2014). The final dataset consisted of 63 samples. After adjustment of the sequence length according to available GenBank data and after deletion of ambiguously aligned positions, the final alignment consisted of 397 bp. The GTR+I+G model was selected as the best-fitting model of nucleotide evolution and employed for maximum likelihood and Bayesian phylogenetic inference.

## Results

**Figure 2.**
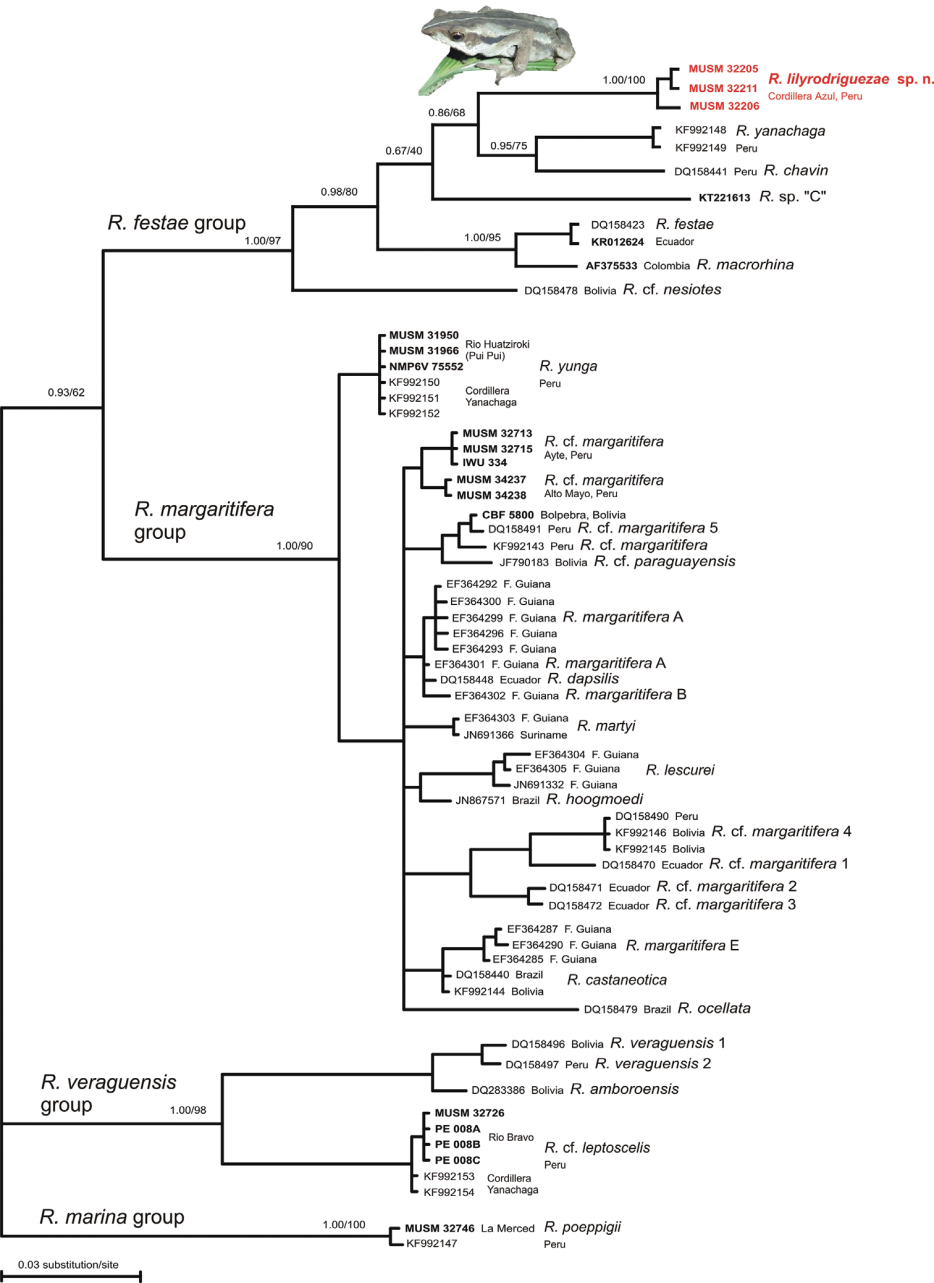
The Bayesian consensus tree resulting from analysis of the mitochondrial 16S rRNA gene dataset for South American *Rhinella* species based on the data from Moravec et al. (2014) with a special focus on the *R. festae* group. New samples are in bold. Bayesian posterior probabilities/maximum likelihood bootstrap values for the main clades (species groups) and within the *R. festae* group are indicated above each node. Three specimens of the new species collected in the Cordillera Azul National Park, Peru, are in red.

**Phylogenetic analysis and systematics.** The maximum likelihood (ML) analysis and Bayesian phylogenetic inference produced trees with the same topologies and strong support for the main clades. Our phylogenetic tree supported the *R. festae* group (Bayesian posterior probabilities 1.00/ML bootstrap 97) and the distinctiveness of the morphologically identified new species of *Rhinella* from the Cordillera Azul National Park. The new species was most closely related to a sister group composed of *R. yanachaga* and *R. chavin* (Fig. 2), both from mountains in central Peru. In the *R. margaritifera* group, our analysis also showed that three new samples of the recently described *R. yunga* (MUSM 31950, 31966, NMP6V 75552) from Rio Huatziroki, a buffer zone of the Protected Forest Pui Pui, Junín, Peru, are genetically identical to the type material (Fig. 2). In addition, our analysis inferred one new, yet unnamed lineage (a candidate species) of the *Rhinella margaritifera* species group from Ayte, Junín (Cordillera Central) and Alto Mayo, San Martín (Cordillera Oriental) in the Peruvian Amazonia. Rhinella cf. margaritifera from Bolpebra (Bolivia) is nested together with two samples from Peru and one Bolivian sample of R. cf. paraguayensis. Within the *R. veraguensis* group, four new samples of R. cf. leptoscelis (an adult specimen MUSM 32726 and conspecific torrenticolous tadpoles PE 008A, PE 008B, PE 008C) from Rio Bravo, Protected Forest Pui Pui, Junín, Peru proved to be closely related to two published sequences from Cordillera Yanachaga, Pasco, Peru (KF992153, KF992154).

### Rhinella lilyrodriguezae sp. n.

Taxon classificationAnimaliaAnuraBufonidae

http://zoobank.org/8FD9CFFD-7311-42A5-85E2-2AB00CF4F7E7

Rhinella cf. festae : Tasker and Twomey 2015: Pag. 1, figs 7–9.

#### Suggested English name.

Lily Rodriguez’s Beaked Toad

#### Suggested Spanish name.

Sapo picudo de Lily Rodríguez

#### Holotype.

**Figure 3.**
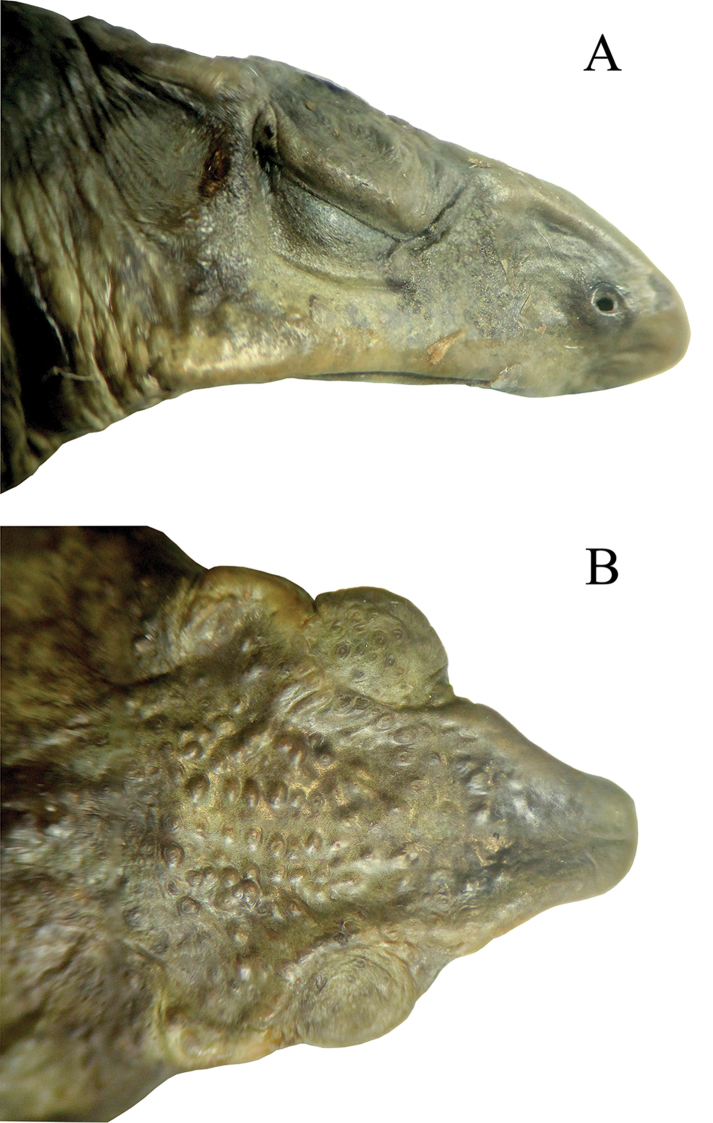
Holotype of *Rhinella lilyrodriguezae* sp. n. (MUSM 32204, head length = 15.9 mm), **A** lateral, and **B** dorsal views of head. Photos by J. C. Cusi.

**Figure 4.**
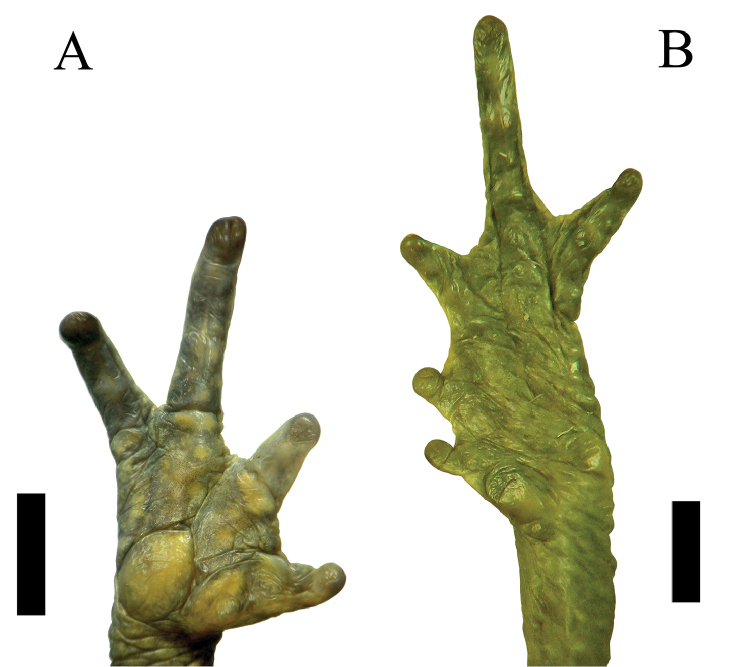
Holotype of *Rhinella lilyrodriguezae* sp. n. (MUSM 32204), **A** palmar, and **B** plantar views of right hand and foot. Scale bar 5 mm. Photos by Juan C. Cusi.

**Figure 5.**
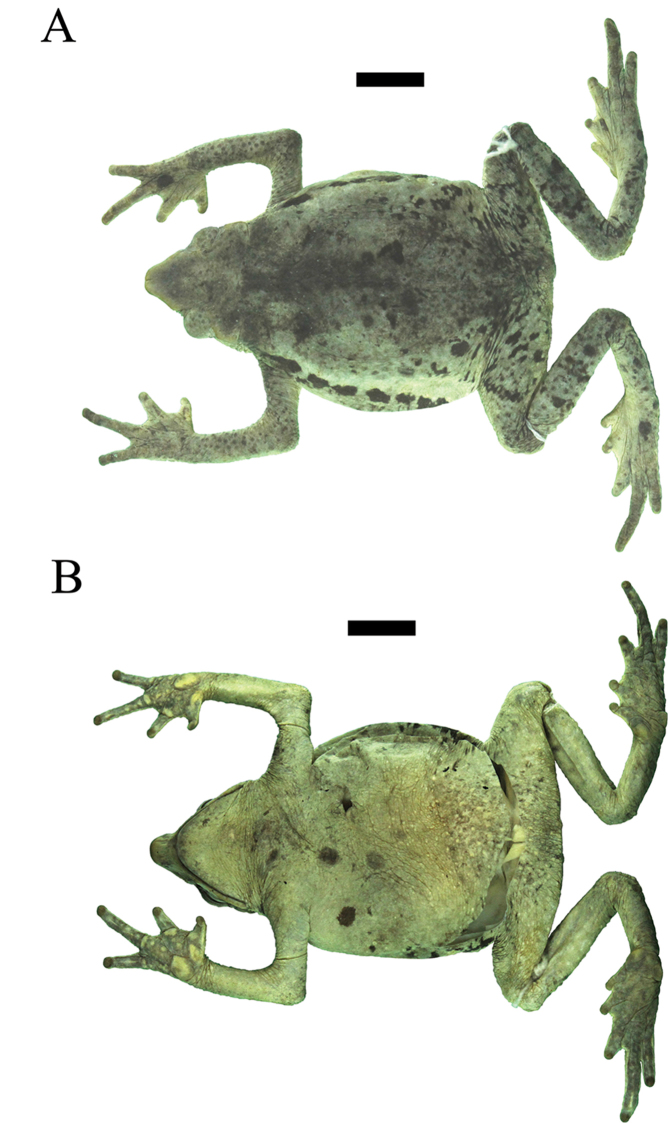
Holotype of *Rhinella lilyrodriguezae* sp. n. (MUSM 32204) in alcohol, **A** dorsal, and **B** ventral views. Scale bar 10 mm. Photos by J. C. Cusi.

**Figure 6.**
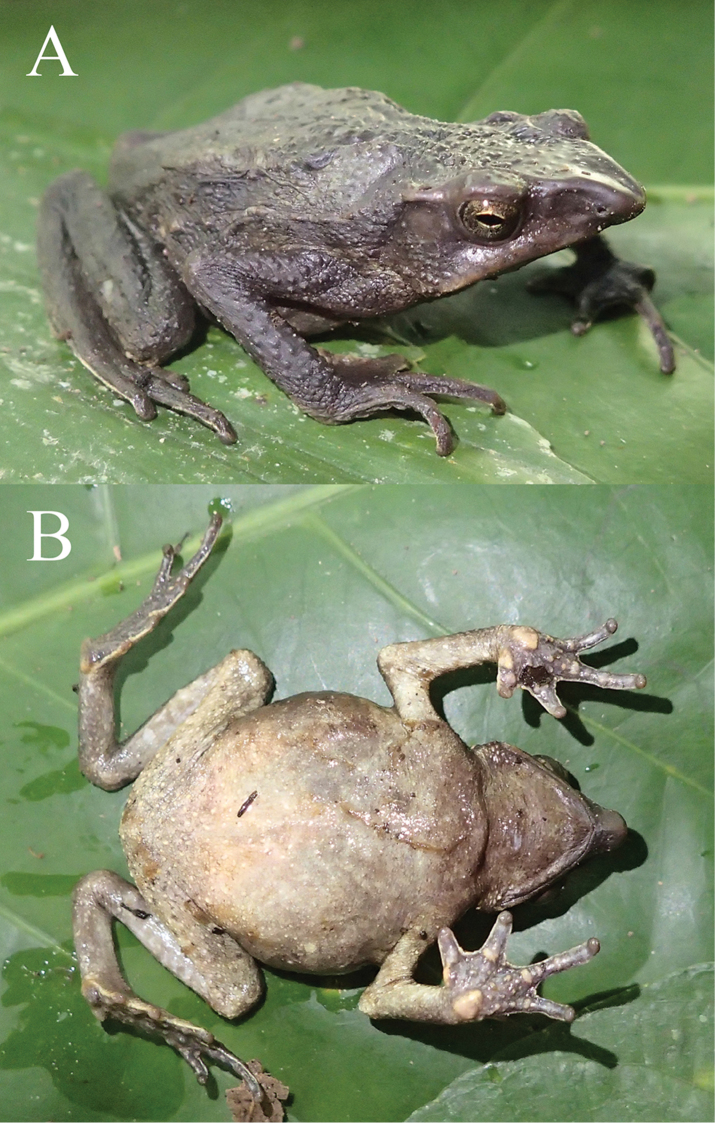
Holotype of *Rhinella lilyrodriguezae* sp. n. (MUSM 32204, SVL = 58.3 mm) in life. **A** Laterodorsal and **B** ventral views of the coloration during the day. Photos by K. Tasker.

MUSM 32204 (field number KT 75, Figs 3–6). An adult gravid female collected *ca.* 20 km from Park Rangers Center N° 53 “Shapaja” of the Cordillera Azul National Park (08°11'15.1"S, 76°12'36.8"W, 1260 m), Alto Biavo District, Bellavista Province, San Martín Region, Peru, collected on 27 September 2013 by K. Tasker and J. C. Cusi.

#### Paratypes.

**Figure 7.**
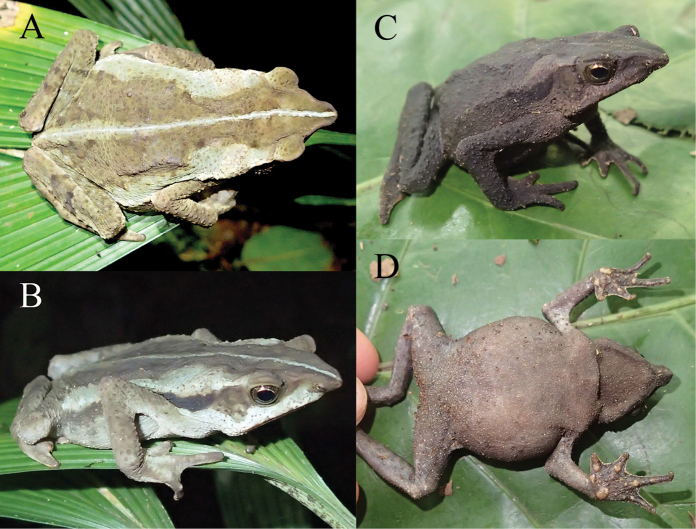
Paratype of *Rhinella lilyrodriguezae* sp. n. (MUSM 32201, SVL = 57.5 mm) in life. **A** Dorsal and **B** laterodorsal views of the coloration at night **C** Laterodorsal and **D** ventral views of the coloration during the day. Photos by K. Tasker.

**Figure 8.**
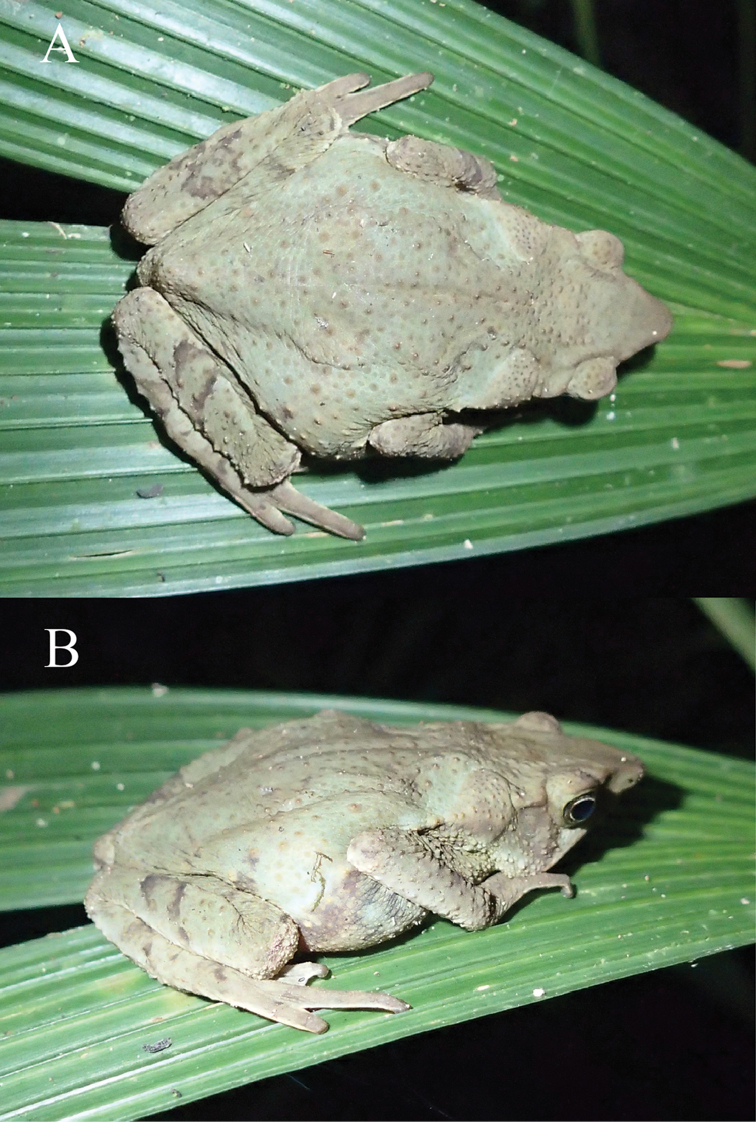
Paratype of *Rhinella lilyrodriguezae* sp. n. (MUSM 32205, SVL = 47.1 mm) in life. **A** Dorsal and **B** laterodorsal views of the coloration during the day. Photos by K. Tasker.

Five individuals (Figs 7–8): an adult female MUSM 32206 (field number KT 76), two juveniles MUSM 32211 (field number KT 84), 32213 (field number KT 72) collected with the holotype; two adult females MUSM 32201 (field number KT 89), MUSM 32205 (field number KT 87), same locality as holotype, collected on 28 September 2013 by K. Tasker and J. C. Cusi.

#### Diagnosis.

A large species of the *Rhinella festae* group confirmed by *16S* DNA barcoding. The new species can be diagnosed by the following combination of characters: (1) large size, SVL 47.1–58.3 mm in females (n = 4), males are unknown; (2) eight presacral vertebrae; (3) sacral vertebrae fused with coccyx; (4) snout long, acuminate, pointed to rounded terminally in dorsal view; snout protuberant, directed slightly anteroventral in profile as a “shark snout”; (5) cranial crests moderately developed; (6) canthal, supraorbital, postorbital and supratympanic crests continuous, distinctly elevated in female, slightly elevated in juveniles; pretympanic crest present, occipital crest absent; (7) tympanic membrane present, tympanic annulus weakly defined; (8) mandibular angle not protruding; (9) parotoid glands moderately large, roughly triangular to rounded in outline, slightly swollen laterally, incorporated into lateral row of tubercles; (10) dorsolateral rows of small, conical tubercles extending from parotoid gland to groin; (11) hands and feet with long digits, fingers basally webbed and toes moderately webbed; (12) skin on dorsum smooth with scattered conical tubercles in females; (13) subarticular tubercles diffuse, round to ovoid; (14) supernumerary tubercles present, round but poorly developed; (15) cloacal sheath absent; (16) in life, dorsum light brown to greenish brown with irregular brown, dark brown or black markings; with or without grey white middorsal stripe; venter cream yellow to brownish grey with minute light cream spots; iris silvery greenish with irregular black mottling.

#### Comparisons with other species.

The new species shares similarities with members of the *Rhinella festae* and *R. acrolopha* groups. Three species of the *R. acrolopha* group from Colombia and Panama (*R. truebae*, *R. lindae* and *R. tenrec*) are large-sized toads similarly to *R. lilyrodriguezae* sp. n. (maximum SVL 65.9 mm in *R. truebae*, 62.2 mm in *R. lindae*, 60.8 mm in *R. tenrec* and 58.3 mm in *R. lilyrodriguezae*). The new species is most similar morphologically to *R. truebae* and *R. tenrec*, but is distinguished by lacking the occipital crest (which is low in *R. truebae* and *R. tenrec*). *Rhinella lilyrodriguezae* sp. n. can also be distinguished from *R. tenrec* by having a sacrum fused with the coccyx, cranial crests moderately developed and tympanic membrane present (sacrum not fused with the coccyx, cranial crests poorly developed, tympanic membrane absent in *R. tenrec*); from *R. truebae* and *R. lindae* by having a dorsolateral row of small conical tubercles extending from parotoid gland to groin, hands basally webbed, and feet moderately webbed (dorsolateral fold formed by the tubercles fusion, hands and feet extensively webbed in *R. truebae* and *R. lindae*). *Rhinella lindae* possesses a snout slightly directed upwards (snout directed slightly anteroventral as “shark snout” in *R. lilyrodriguezae*).

Other species of the *Rhinella acrolopha* group are *R. acrolopha*, *R. nicefori*, *R. paraguas*, and *R. ruizi*. These species are differentiated from *R. lilyrodriguezae* sp. n. (characters in parentheses) by the absence of the tympanic membrane (present), hands and feet extensively webbed in *R. paraguas* and *R. ruizi*, and reduced webbing in *R. acrolopha* and *R. nicefori* (hands basally webbed and feet moderately webbed), sacrum not fused with the coccyx except in *R. paraguas* (fused), seven presacral vertebrae except in *R. paraguas* (eight) and the presence of occipital crest except in *R. paraguas* (absent). *Rhinella nicefori* and *R. ruizi* have hands and feet with short digits (long digits). *Rhinella paraguas* and *R. ruizi* have cranial crests very low (moderately developed). *Rhinella nicefori* has a dorsolateral row of enlarged tubercles extending from the posterior margin of the parotoid gland to a point about three-fourths the distance between the axilla and groin (conical tubercles extending to groin). *Rhinella acrolopha* possesses a snout directed markedly anteroventrally (directed slightly anteroventral as “shark snout”) and a dorsolateral row of depressed tubercles extending from the posterior margin of the parotoid gland posteriorly to a point about two-thirds distance between axilla and groin (conical tubercles extending from parotoid gland to groin).

The remaining species of the *R. acrolopha* and *R. festae* groups from Colombia and Ecuador (*R. festae*, *R. macrorhina* and *R. rostrata*) are distinguished from *R. lilyrodriguezae* sp. n. by lacking the tympanic membrane (present), by having hands and feet extensively webbed (hands basally webbed and feet moderately webbed), seven presacral vertebrae (except for *R. macrorhina* which has eight), snout directed markedly anteroventrally in *R. festae* and *R. macrorhina* and straight in *R. rostrata* (directed slightly anteroventral). *Rhinella festae* and *R. macrorhina* have occipital crest well developed (absent). *Rhinella macrorhina* is distinct in having a sacrum not fused with the coccyx (fused), and a dorsolateral row of small tubercles extending from posterior margin of parotoid gland posteriorly to a point about one-half distance between axilla and groin (tubercles extending from parotoid gland to groin). *Rhinella festae* possesses dorsolateral row of slightly enlarged, conical tubercles extending from posterior margin of skull to a point about three-fourths distance between axilla and groin (tubercles extending from parotoid gland to groin) and hands with short digits (long digits).

Peruvian species of the *R. festae* group (*R. chavin*, *R. manu*, *R. nesiotes* and *R. yanachaga*) are distinguished from *R. lilyrodriguezae* sp. n. by having smaller females (maximum SVL 21.4 mm in *R. manu*, 23.6 mm in *R. nesiotes*, 45.7 mm in *R. yanachaga*, except in *R. chavin* with 54.8 mm; vs. 58.3 mm in *R. lilyrodriguezae*) and by having webbing of hands and feet fleshy (membranous in *R. lilyrodriguezae*). *Rhinella chavin* possesses a snout rounded in lateral view (snout protuberant, directed slightly anteroventral as “shark snout”), large [about twice ED], ovoid parotoid glands (moderately large [about same size as ED] and triangular), dorsolateral row of large, nearly round elevated tubercles beginning above insertion of forelimb extending to inguinal region (small, conical tubercles extending from parotoid gland to groin), elevated, elongate glands on forearm, tibia and outer dorsal margin of the foot and hand (glands absent) and hands and feet with relatively short digits (long digits). *Rhinella nesiotes* has a snout rounded in lateral view (snout protuberant, directed slightly anteroventral as “shark snout”), lacks cranial crests (moderately developed), low ovoid parotoid glands (moderately large and triangular), lacks dorsolateral row of tubercles (present) and hands and feet with relatively short digits (long digits). *Rhinella manu* has a snout pointed in lateral view (snout protuberant, directed slightly anteroventral as “shark snout”), large [about twice ED], oblong parotoid glands to the point of being nearly spherical (moderately large [about same size as ED] and triangular) and inconspicuous cranial crests (moderately developed). *Rhinella yanachaga* is most similar to *R. lilyrodriguezae* sp. n. from cloud forests of central Peru, both have cranial crests, tympanic membrane distinct, moderately large [about same size as ED] parotoid glands, dorsolateral rows of small, conical tubercles extending from parotoid gland to groin, fingers and toes relatively long and long, slender extremities. Nevertheless, *R. yanachaga* differs from *R. lilyrodriguezae* sp. n. by having a snout slightly protruding in lateral view (snout protuberant, directed slightly anteroventral as “shark snout”), well developed webbing on hands and feet (hands basally webbed, and feet moderately webbed), dorsal skin smooth without keratin-tipped tubercles in females (dorsum smooth with scattered conical tubercles in females) and dorsal coloration in life is dark brown with small, irregular, green spots and markings (dorsum light brown to greenish brown with irregular brown, dark brown or black markings).

#### Description of the holotype.

Gravid female; body robust; SVL 58.3 mm; head triangular in dorsal view; head wider than long (HW 1.12 times HL), head width 30% of SVL; head length 27% of SVL; head narrower than body; snout acuminate, rounded terminally in dorsal view; not bulbous at tip; distance from the nostril to the tip of the snout (3.2 mm) is noticeably less than the distance from the nostril to the eye (5.8 mm), constituting 20% of head length; snout long, protuberant, directed slightly anteroventral as “shark snout” in profile (Fig. 3); snout with a ventral keel; canthus rostralis angular, rounded in lateral view; loreal region concave; nostrils small, rounded, not protruding, directed laterally, beyond anterior margin of lower jaw; internarial area concave; eye diameter equal to half the interorbital distance (ED/IOD = 0.5), ED noticeably shorter than E-D; canthal ridges angular evident; cephalic crests moderately developed; pre-, supra-, postorbital crests distinct, continuous; occipital crest absent; supratympanic crest evident, expanded laterally; pretympanic crests well defined; tympanic annulus weakly defined, superficial tympanic membrane present, not in contact with parotoid glands or postorbital crests; tympanum diameter smaller than eye diameter; parotoid glands moderately large (about same size as ED), roughly triangular in outline; upper eyelid covered with many, low, keratin-tipped tubercles; dorsal and lateral surfaces of head bearing many warts; forearms long, slender; forearm length 26% of SVL; dorsal surface of forelimbs spiculate, bearing densely scattered subconical tubercles; hand length 28% of SVL; hands with long fingers; relative lengths of fingers I < II < IV < III; finger tips rounded; fingers basally webbed, extended between fingers II–IV; Finger IV bears well-defined lateral fringes (Fig. 4A); palmar tubercle prominent, round, larger than oval thenar tubercle, about one half size of the palmar tubercle; subarticular tubercles diffuse, low, round to ovoid; supernumerary tubercles low, poorly developed, indistinct; hindlimbs long, slim; tibia length 40% of SVL; tibia longer than foot; dorsal surface of hindlimbs spiculate with subconical tubercles; foot length 39% of SVL; toes long; relative lengths of toes I < II < III < V < IV; toes tips rounded; toes moderately webbed, with the following formula: I 1–2- II 1-–2 III 1–3^+^ IV 3^+^–2 V; free portions of all toes bear well-defined lateral fringes (Fig. 4B); tarsal fold absent; inner metatarsal tubercle large, slightly elliptical, weakly protuberant; outer metatarsal tubercle round, smaller than inner metatarsal tubercle, half the size of inner metatarsal tubercle; subarticular tubercles diffuse, low, round to ovoid; supernumerary tubercles indistinct, skin on dorsal surface of the body with numerous small, round, elevated tubercles, bearing single keratinized tip; flanks with lower density of tubercles than dorsum; dorsolateral row of small, conical tubercles extending from parotoid gland to groin, not forming a distinct dorsolateral fold; skin of venter and throat, chest and venter granular; cloacal opening directed posteriorly at the mid-level of the thighs); tongue narrow, about 2.5 times as long as wide, not notched posteriorly, posterior one half free; choanae small, ovoid, widely separated and partially concealed by palatal shelf of maxilla.

#### Measurements (in mm) of the holotype.

SVL: 58.3; HW: 17.8; HL: 15.9; ED: 4.4; IOD: 9.3; EW: 3.9; EL: 6.4; IND: 4.3; E–N: 5.8; NSD: 3.2; SL: 9.1; FL: 15.4; HNDL: 16.3; FEML: 23.7; TL: 23.4; FOOTL: 22.6.

#### Coloration of the holotype in alcohol

**(Fig. 5).** General dorsal coloration light brown; dorsum and hind limbs with small, irregular, dark brown spots and markings; flanks cream, with a discontinuous, well defined, broad, black ventrolateral band beginning behind tympanum and extending to inguinal region; dark grey transverse bars on shanks; tympanum brown; upper lip cream without bars or spots; dorsolateral row of tubercles reddish brown, sharply contrasting with the black discontinuous ventrolateral band; throat, chest and venter gray with minute light cream spots; ventral surfaces of thighs gray with minute dark grey spots; ventral surfaces of hands and feet dark gray; subarticular and supernumerary tubercles cream on hands.

#### Coloration of holotype in life

**(Fig. 6).** Diurnal coloration of dorsum and flanks dark brown; flanks with irregular, dark green dorsolateral blotches extending from behind the parotoid glands to sacral region; dorsolateral row of tubercles light brown; throat dark gray; chest and belly grey with minute light cream spots extending to the thighs; lower side of the belly cream yellow; iris silvery greenish with irregular black mottling.

#### Variation

**Figure 9.**
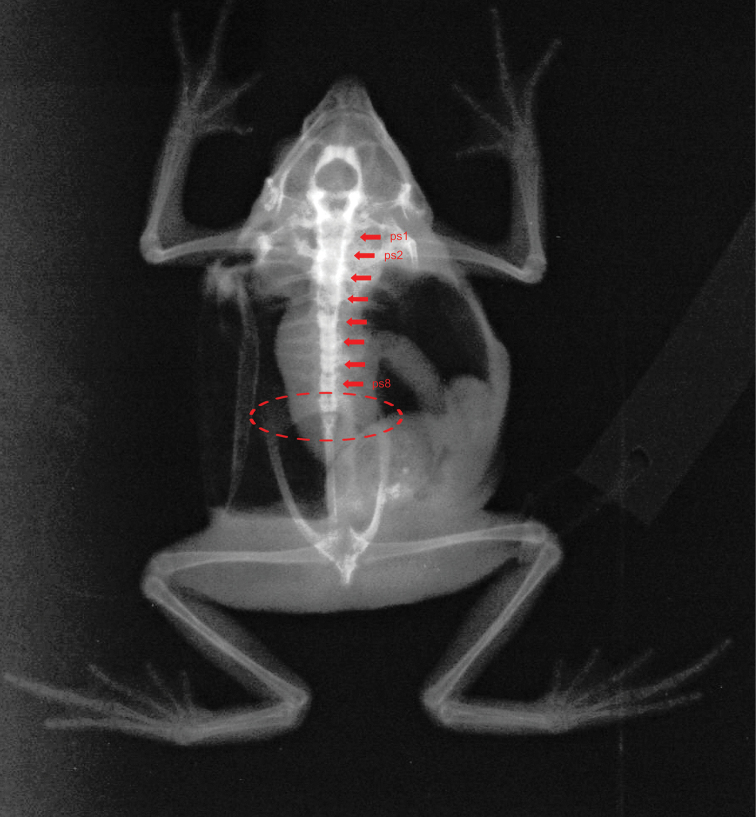
X-ray of the paratye *Rhinella lilyrodriguezae* sp. n. MUSM 32206. Photos by J. C. Cusi.

**(Figs 7–9).** Considerable variation between nocturnal and diurnal coloration was observed. The nocturnal coloration of an adult female (MUSM 32201, Fig. 7 A, B) was described as follows: dorsum light brown dorsally; whitish grey middorsal stripe extending from tip of the snout to cloaca; light cream parotoid glands; broad, whitish grey dorsolateral stripe on each side of flanks extending from behind the eyelids to groin; flanks with continuous, broad, black ventrolateral band extending from tympanic posterior region to groin; tip of the snout, eyes, eyelids, crown of head and tympanum light brown; upper lip, angle of the jaws and ventrolateral region of the flanks is whitish grey; dorsolateral row of tubercles reddish brown just above the black ventrolateral band; forelimbs and hands are brown, dorsum of the hands with whitish grey blotches in direction to the fingers; hindlimbs brown with transversal dark brown bars. The diurnal coloration of the same specimen (Fig. 7C, D): dorsum and flanks dark brown; gray middorsal stripe from tip of the snout to cloaca; parotoid glands cream brown; venter brownish grey marbled with cream; throat darker brown. An adult female (MUSM 32205; Fig. 8) lacked a middorsal stripe and had flanks with irregular dark grey spots at night. During the day, the same specimen was characterized by a dark green dorsolateral stripe on each side of body extending from parotoid gland to groin; venter brownish grey marbled with cream; irregular dark grey spots on thighs, lateral and lower side of the belly; white blotches on middle area of the belly. Two females (MUSM 32206, 32201) were of similar coloration as MUSM 32205.

The remaining paratypes show some variation in nocturnal color pattern. The overall dorsal coloration of the juveniles (MUSM 32211, 32213) is light grey with darker irregular markings forming a “dead-leaf pattern” from between eyes to cloacal region. Cranial crests are more prominent in adults than in juveniles. All specimens, except the holotype, have tip of snout acuminate in dorsal view. Radiographs show that all specimens have eight presacral vertebrae and a sacrococcygeal articulation (Fig. 9). For variation in measurements see Table 2.

**Table 2.** Morphometric measurements in mm of the holotype and paratypes of *Rhinella lilyrodriguezae* sp. n. See text for abbreviations.

Measurements	Holotype	Paratypes				
	MUSM 32204	MUSM 32201	MUSM 32206	MUSM 32205	MUSM 32211	MUSM 32213
**Sex**	Female	Female	Female	Female	Juvenile	Juvenile
**SVL**	58.3	57.5	55.5	47.1	34.9	27.8
**HW**	17.8	18.0	17.2	11.4	11.6	9.4
**HL**	15.9	15.1	14.1	8.9	9.0	9.2
**ED**	4.4	4.3	4.3	4.2	3.7	3.2
**IOD**	9.3	8.8	8.8	8.4	5.6	4.9
**EW**	3.9	3.9	3.9	3.7	3.4	2.8
**EL**	6.4	6.8	6.0	6.2	3.9	3.2
**IND**	4.3	4.4	4.3	4.1	2.5	2.1
**E-N**	5.8	5.7	5.6	5.5	3.4	3.9
**NSD**	3.2	3.2	3.0	2.9	1.7	1.3
**SL**	9.1	9.0	8.7	8.1	5.8	4.9
**FL**	15.4	15.2	14.1	13.5	9.1	7.2
**HNDL**	16.3	16.4	15.1	13.7	8.7	6.5
**FEML**	23.7	23.5	22.0	20.3	13.2	11.7
**TL**	23.4	23.2	21.4	19.9	13.1	11.3
**FOOTL**	22.6	23.3	22.0	20.9	13.5	10.8

#### Etymology.

The specific epithet *lilyrodriguezae* is a noun in the genitive case and a patronym for Dr. Lily Rodriguez, for her contributions to the knowledge of the Peruvian amphibians and her initiatives that have promoted the creation of numerous natural protected areas in Peru, such as the Cordillera Azul National Park.

#### Distribution, ecology and conservation status.

*Rhinella lilyrodriguezae* sp. n. is only known from the Shapaja sector within the CAZNP in northern Peru, at elevations between 1245 and 1280 m a.s.l. (Fig. 1). It was encountered in montane forest during the dry season. The type locality was accessed by a hike of 7 hours across a small trail from the Misterioso River (natural boundary between natural protected area and a forest concession dedicated to wood extraction). The species has nocturnal and semiarboreal mode of life (all individuals were found at night between 20:33 and 22:49 on leaves of bushes between 10 and 100 cm above the ground, along a small creek). A negative impact of logging, soil removal and noise pollution was observed around the localities of the new species. Subsistence game hunting and exaggerated fishing by local population are other factors threatening the local biodiversity.

One gravid female (MUSM 32204, SVL 58.3 mm, holotype) contained 185 ovarian eggs (left ovary: 95; right ovary: 90) with an average diameter of 2.8 ± 0.2 mm (3.2–2.7 mm, n = 20), which are pale cream yellow in preservative. The presence of numerous, large, pigmented eggs and association of the individuals with water bodies suggest endotrophic larvae (e.g., direct development or nonfeeding tadpoles that develop in water or moist soil; Peloso et al. 2014), as can be expected in *R. manu*, *R. paraguas* and *R. tacana* (Grant and Bolívar-G. 2014, Catenazzi pers. comm.) The call of *Rhinella lilyrodriguezae* was not recorded. Other anuran species that occur in the area of the type locality include Hyloscirtus cf. phyllognathus, *Pristimantis peruvianus*, *P. ventrimarmoratus*, Rulyrana cf. flavopunctata, and *Osteocephalus mimeticus* (juveniles). We classify *Rhinella lilyrodriguezae* sp. n. as “Data Deficient” according to the IUCN red list criteria (IUCN 2016) based on the limited information on its geographic range.

## Discussion

An early comprehensive revision of the former genus *Rhamphophryne* (= *Rhinella acrolopha* group) based on external morphology and osteology hypothesized multiple evolutionary histories and complex morphological variation within the genus (Trueb 1971). Knowledge of the phylogenetic relationships among most of the species of the *R. acrolopha* group remains largely unknown mainly due to few collected specimens (*R. truebae*, n = 1; *R. lindae*, n = 2; *R. nicefori* n = 5; *R. tenrec*, n = 11; Trueb 1971, Rivero and Castaño 1990, Lynch and Renjifo 1990), absence of available genetic material, and the fact that several species seem to be seriously threatened (e.g., *R. rostrata* is possibly extinct in the wild, Bolívar and Lynch 2004, Stuart et al. 2008).

Our 16S rRNA analysis supports the monophyly of the *Rhinella festae* clade of Moravec et al. (2014) and confirms the inclusion of species previously assigned to this group. Furthermore, our analysis adds *R. lilyrodriguezae* sp. n. and *Rhinella* sp. C to the *R. festae* group. Although we did not include *R. manu* in our tree (due to the unavailability of 16S rRNA sequence data), its incorporation into the *R. festae* group is highly likely; as already showed by Chaparro et al. (2007) in one clade comprised by [[*R. festae* + *R. chavin*] + [*R. manu* + *R. nesiotes*]] using combined mitochondrial and nuclear DNA data. On the other hand, the species *R. rostrata* and *R. nesiotes* are tentatively removed from the *festae* group (sensu Moravec et al. 2014) due to absence of available genetic material that confirms their inclusion into this group. The specimen UTA 53310 “*R. nesiotes*” from Serranía de Bella Vista, Bolivia used by Pramuk (2006), Chaparro et al. (2007), Pramuk et al. (2008) and Moravec et al. (2014), is now identified as Rhinella cf. nesiotes to occur at about 1000 km south of the type locality of *Rhinella nesiotes*, which inhabits in an isolated mountain ridge between the Pachitea and Ucayali rivers, Central Peru. This identification is justified on the basis of geographic distance, the scarce knowledge of the distribution range of the species and the occurrence of species morphologically similar in the Bolivian area as *R. tacana*, possibly confused with it (Fig. 1). In absence of molecular data, we cannot assign *Rhinella nesiotes*, known from four specimens collected at Serranía El Sira in Peru, to any recognized groups. Furthermore, a morphological examination of Bolivian material and comparison with Peruvian specimens of *R. nesiotes* is required. Thus, according to our current knowledge, the *Rhinella festae* group contains the following eight species: *R. chavin*, *R. festae*, *R. lilyrodriguezae* sp. n., *R. macrorhina*, *R. manu*, *R. yanachaga*, R. cf. nesiotes and *Rhinella* sp. C. The recently described *R. tacana* extended from Serranía Eslabon, northern Bolivia to Urubamba River Basin, southern Peru (Padial et al. 2006, Chávez et al. 2013) is morphologically most similar to *R. manu*, and is expected to be part of the *festae* group. The *Rhinella festae* group represents a morphologically and genetically diverse group of toads broadly distributed in the region of eastern slopes of the Cordillera Occidental from Colombia, upper Amazon Basin and lower Andean slopes of Ecuador and eastern slopes of the Cordillera Oriental from Peru and Bolivia. Our phylogenetic analysis supports the unique position of *R. lilyrodriguezae* sp. n., which is distributed in montane forests of the Cordillera Oriental, San Martín, northern Peru. *Rhinella lilyrodriguezae* sp. n. is a semiarboreal species because it was observed climbing on leaves and branches (approximately 1 m above ground) close to lotic aquatic bodies of medium-size and moderate water flow, and dwelling on the ground during the day. All species of the *R. festae* and *R. acrolopha* groups display diverse types of habitat use. Some species are semiarboreal (*R. chavin*, *R. lilyrodriguezae* sp. n., *R. nesiotes*, *R. paraguas*) or arboreal (*R. manu*, *R. yanachaga*), others are terrestrial (*R. festae*, *R. macrorhina*, *R. rostrata*). Most members of both groups inhabit rainforests and montane forests at elevations between 1200 m and 3600 m, except for *R. tenrec* occurring in the Chocó lowlands, and *R. festae* inhabiting the Amazonian lowlands.

Finally, the monophyly of the *Rhinella festae* clade as proposed by Moravec et al. (2014) is further supported by our present data, and species composition is modified by inclusion of additional taxa previously assigned to the *R. acrolopha* group (i.e. *R. macrorhina* and *Rhinella* sp. C), as supported by high morphological similarity and molecular affinity. However, additional molecular data are required for any conclusive observations. Our study constitutes a starting point for understanding the diversification within this radiation. Additional morphological and molecular evidence from a larger number of taxa is needed to reach a better knowledge of the evolution, systematics and biogeography of this interesting South American lineage of bufonid anurans.

## Supplementary Material

XML Treatment for Rhinella lilyrodriguezae

## Appendix

**Specimens examined**

*Rhinella chavin* (9 specimens). PERU: HUÁNUCO, Pachitea, Chaglla, Palma Pampa, 20 km southeast of Chaglla, 3010 m: MUSM 20028 (holotype), MUSM 18439–18446.

*Rhinella manu* (8 specimens). PERU: CUSCO: Paucartambo, Kosñipata: MUSM 21129, 27931, 30385; Rocotal: MUSM 26282, 27932; Mirador Tres Cruces: MUSM 27929, 27933; San Pedro: MUSM 27930.

*Rhinella nesiotes* (4 specimens). PERU: HUANUCO, Puerto Inca, Yuyapichis, Comunidad El Sira: MUSM 29386, 29387, 29390, 29404.

*Rhinella yanachaga* (4 specimens). PERU: PASCO: Oxapampa, Oxapampa, Yanachaga-Chemillén National Park (PNYCH), W side of the Cordillera Yanachaga near Río San Alberto, 2600 m: MUSM 19994 (holotype); village San Alberto: MUSM 33309-33310; Buffer zone of the PNYCH: MUSM 33520.
